# Observations on Gangrenes and Mortifications, Accompanied with, or Occasioned by, Convulsive Spasms, or Arising from Local Injury, Producing Irritation

**Published:** 1790

**Authors:** Charles White

**Affiliations:** Surgeon to the Infirmary and Lunatic Hospital, and Vice-President of the Literary and Philosophical Society at Manchester, &c.


VIII. *Observations on Gangrenes and Mortifications, accompanied with, or occasioned by, convulsive Spasms, or arising from local Injury, producing Irritation.* *By* Charles White, *Esq. F. R. S. Surgeon to the Infirmary and Lunatic Hospital, and Vice-President of the Literary and Philosophical Society at Manchester,* &c. 8vo. Warrington, 1790.

FOR some time after the Peruvian bark was first recommended in the cure of gangrene, it was considered as a certain remedy in every affection of that sort; but although in many of these it has been found very powerful and efficacious, there have been others in which it has been exhibited with less success, or has altogether failed. These failures have led some men of great eminence in practice to place less reliance on its virtues than it seems really to deserve, and to be even doubtful if it has any well-founded claim to a preference to cordials in general. This has probably been owing to its its having been given indiſcriminately in all caſes, notwithſtanding they aroſe from a variety of cauſes totally different, and which naturally required very different treatment.

That the bark will not ſtop all mortifications that may be, and have been, ſtopped by other remedies is evident from the many trials that have been made, both of that medicine and opium, in that particular ſpecies which attacks the feet and toes, and which, though ſlow in its progreſs, is accompanied with local pain or general uneaſineſs. The Public is under great obligations to the late Mr. Pott for the criterions by which this ſpecies of mortification may be diſtinguiſhed with preciſion, and the recommendation of large and repeated doſes of opium as its mode of cure.

The ingenious and reſpectable author of the work before us flatters himſelf he has found out alſo a remedy for another ſpecies of gangrene, which is the ſubject of the preſent eſſay; but notwithſtanding this remedy, as well as that of opium, juſt now alluded to, may ſtand the teſt of experience and time, the bark, he candidly obſerves, will ſtill be uſeful in mortifications occaſioned by a relaxed habit, by a broken and diſſolved craſis of the blood, and in thoſe ariſing from a kind of acrimony attended with putreſcency. And in caſes highly inflammatory, bleeding, he adds, and the antiphlogiſtic plan will be neceſſary, together with nitre and the mineral acids.

In general, this concluſion, he remarks, may be ſafely drawn, that gangrene, ariſing from different cauſes, in different habits, and under particular circumſtances, requires different methods of treatment; as, in general, all the different ſpecies of a diſorder, ranged under one head, will not, at all times, and in all caſes, be cured by one and the ſame medicine, though curable by others.

The particular ſpecies of mortification which is the ſubject of the pamphlet before us is that accompanied with, or occaſioned by, convulſive ſpaſms, or ariſing from local injury, producing irritation, which is alſo of the ſpaſmodic kind. The remedy Mr. White recommends for this is a large and frequently repeated doſe of muſk and ſalt of hartſhorn, the uſe of which, he tells us, he diſcovered rather unexpectedly and by accident. Muſk and the volatile alkali, he allows, have been given before his time; but he does not find that they have, generally, been preſcribed in ſufficient quantities, or ever

 admiadministered in this species of mortification; so that hitherto the full extent of their virtues, especially in this complaint, has not been observed or ascertained.

Our author is well aware of the uncertainty of ascertaining medical facts, especially when those facts are not very numerous; and he also knows that mortifications will frequently proceed to a certain point and then stop, without much assistance; as if the disorder had come to a crisis, and nature, after having thrown all the morbid matter on one part, had, by her own exertions, relieved herself. In mortification, he observes, arising from cold, it is not very uncommon to see the complaint stop as soon as the cause is removed.

When he first employed this medicine in the complaints to which his present publication relates, it was not from any expectation, he owns, of stopping their immediate progress, but merely to combat disagreeable symptoms, such as the singultus, subsultus tendinum, and other convulsive spasms. He soon found, however, that it not only removed these unpromising appearances, but also procured ease, sleep, and a gentle diaphoresis, whilst, at the same time, the mortification regularly stopped. The circumstance cumſtance ſtruck him, but he ſcarcely ventured to flatter himſelf that it was owing, in the firſt inſtance, to the medicine, till, from repeated trials of it, he obſerved the ſame uniform effects. He aſſures us, that in moſt of the caſes, in this ſpecies of mortification, that have occurred in his practice, it has ſucceeded to the utmoſt of his wiſhes, viz. when the complaint has been accompanied with, or occaſioned by, convulſive ſpaſms, or has ariſen from local injury, producing irritation. He has tried it in gangrene and mortification ariſing from other cauſes, and not attended with thoſe ſymptoms, but has frequently been diſappointed in its effects.

As proofs of its efficacy, Mr. White gives the following account of four of the caſes that occurred during the firſt four years of his giving this medicine.

Many other ſimilar caſes, we are told, have occurred to him ſince, and been attended with the ſame ſucceſs, but theſe he has thought it would be ſuperfluous to deſcribe. On dubious points, as the author very properly obſerves, too many caſes can hardly be produced; but convinced, as he is, of the facts he relates, he has thought it unneceſſary to multiply them, eſpecially as they will receive additional ſtrength from a theſis which his friend, Mr. Darbey, late houſe ſurgeon of the Mancheſter Infirmary, intends to ſupport for a degree in phyſic.

"CASE I.

"On the 13th of September, 1778, I per-

"formed the operation of lithotomy upon Mr.

"Patrick, a gentleman aged ſixty-two, and

"extracted a ſtone weighing between three

"and four ounces. The inflammatory ſymp-

"toms ran very high for two or three of the

"firſt days, attended with great pain and ten-

"ſion of the abdomen. The antiphlogiſtic

"plan was purſued, together with anodyne fo-

"mentations and embrocations to the parts af-

"fected; but the tenſion and pain of the ab-

"domen not giving way, the belly, on the

"fourth day after the operation, was rubbed

"with mercurial ointment, and it was repeated

"twice a day. His pulſe was quick, and his

"tongue dry. On the ſixth a ſingultus came

"on; there was no digeſtion in the wound;

"and the other bad ſymptoms continued. I

"directed four ſpoonfuls of the *julepum e moſ-*

"*cho* to be taken every three hours. On the

"ſeventh he remained much the ſame as the

"preceding

" preceding day. After this, a little digeſtion

" appeared upon the wound; the hiccup was

" leſs frequent; the pain and tenſion of the

" abdomen abated; and all the ſymptoms be-

" came more favourable. I now entertained

" hopes of his recovery till the twenty-ſeventh,

" when he had a violent, cold, ſhivering fit, and

" all the bad ſymptoms returned with greater

" force. His pulſe was one hundred and forty

" in a minute, his ſkin dry, and tongue brown

" and hard, without the leaſt moiſture upon it.

" The hiccup was conſtant; the ſubſultus ten-

" dinum very violent: he was delirious; his

" belly was greatly enlarged, and emphyſema-

" tous; the wound black, and perfectly mor-

" tified. I had not now the leaſt idea of his

" recovery; but, in order to abate the ſingul-

" tus and ſubſultus tendinum, I ordered him

" a bolus, containing ten grains of muſk, with

" the ſame quantity of ſalt of hartſhorn, every

" three hours. He was not able to take more

" than four boluſes in the day, and conſe-

" quently only forty grains of muſk with an

" equal quantity of ſalt of hartſhorn. On the

" next morning, the twenty-eighth, a little

" moiſture appeared upon the ſkin, but, in

" other reſpects, he continued as the day be-

" fore.

"fore. On the twenty-ninth the muſk and "ſalt of hartſhorn were continued in the ſame "quantities, and my expectations were more "favourable. His delirium abated on the "thirty-firſt; and I prevailed on him to take "daily eighty grains of muſk with eighty "grains of ſalt of hartſhorn. On the thirty-"ſecond he was ſenſibly better; his pulſe did "not exceed one hundred in a minute; the "ſkin and tongue were moiſt; the belly began "to ſubſide; and the mortification ſeemed to "be ſtopped. On the thirty-third he conti-"nued to mend, but the eighty grains of muſk "and eighty grains of ſalt of hartſhorn were "perſevered in. On the thirty-fourth he took "one hundred and twenty grains of muſk, and "as much ſalt of hartſhorn.

"All his bad ſymptoms left him on the "thirty-fifth; the mortified parts ſloughed "away, and he gradually recovered. In the "whole of this caſe one ounce and thirty-ſix "grains of muſk were taken, and the ſame "quantity of ſalt of hartſhorn.

"CASE II.

"James Ogden, of Mancheſter, aged forty-"ſix, was, on the 6th of March, 1780, re-

"ceived

"ceived into the Manchester Infirmary for a

"compound fracture of the leg. It soon be-

"came inflamed; swelled considerably, with

"much tension; shewed little signs of diges-

"tion; and on the fifth day began to grow

"livid. The bark was given in considerable

"quantities; but notwithstanding the use of

"it, the livid parts were completely mortified.

"His pulse beat one hundred and forty in a

"minute; his skin was hot and dry, and his

"tongue, without moisture, brown and hard.

"A singultus and subsultus tendinum came

"on; he had a wildness in his looks, succeed-

"ed by a delirium. On the eighth day from

"the accident I directed him to take a bolus,

"containing ten grains of musk, with ten

"grains of salt of hartshorn, made up with

"conserve of roses, every three hours. He

"slept better the following night than he had

"ever done since his misfortune, though he

"had no opiate, and the next morning he was

"in a breathing sweat. His symptoms gra-

"dually abated, but he continued the use of

"the bolus till he had taken two ounces and a

"half of musk, and as much salt of hartshorn.

"The mortification stopped, and the dead

"parts, separating from the living, came

" away in large floughs; but there was fuch a
" confiderable lofs of fubftance, that he was
" not able to leave the Infirmary till the 11th
" of December, upwards of nine months from
" the accident, when he was difcharged, cured,
" with a very ufeful leg.

"CASE III.

" On the 1ft of May, 1782, a maiden lady,
" about forty years of age, and of a very cor-
" pulent habit of body, was feized with cold
" fhiverings, fucceeded by much fever, and an
" eryfipelas in her face. A very fenfible apo-
" thecary, who firft faw her, fent her a purg-
" ing potion, an aperient clyfter, and fome al-
" kaline draughts, with a grain of camphor, to
" be taken with lemon juice in the act of ef-
" fervefcence, which were to be repeated every
" four hours. The next day the purging po-
" tion was again ordered. I faw her on the
" third, and found her in a ftate of delirium,
" with great drowfinefs, fubfultus tendinum,
" attended with naufea, a very quick pulfe,
" and the face much fwelled and livid. I di-
" rected her an aperient apozem, with nitre, to
" be taken occafionally; and a bolus, compofed
" of ten grains of mufk and ten grains of the
" cordial

"cordial confection, to be repeated every five "or fix hours, On the fourth, the naufea and "delirium remaining, I ordered her to take "two or three fpoonfuls of the camphor julep, "made with vinegar inftead of water, frequent- "ly in the day. On the fifth her ftomach was "more fettled, but as fhe difliked the mufk "bolus, it was changed into pills. On the "fixth fhe feemed much better in every re- "fpect; the mufk was omitted, and the bark "was ordered, in draughts, to be taken three "times a day. On the feventh her face grew "more painful, and an anodyne fomentation "was frequently applied to it, which appeared "to procure fome eafe. On the eighth all the "bad fymptoms returned with greater violence. "Her face was more livid, the delirium ran "very high, and the fubfultus tendinum was "very troublefome, with a pulfe of one hun- "dred and thirty-five in the minute. I then "directed her ten grains of mufk, with five "grains of falt of hartfhorn, in pills, to be "taken immediately, and to be repeated as "often as fhe could be prevailed upon to take "them. On the ninth fhe was very fenfibly "relieved, her fleep had been more natural, "and fhe was in a gentle perfpiration. On the

"tenth

" tenth the delirium and ſubſultus tendinum " left her, but the muſk and ſalt of hartſhorn " were continued. On the eleventh the livid- " neſs of the face diſappeared, and every ſymp- " tom became much more favourable; pulſe " one hundred in a minute; but the muſk and " ſalt of hartſhorn were repeated. On the " twelfth, pulſe ninety in a minute, ſhe ad- " vanced very faſt towards recovery, and took " the laſt doſe of muſk that I thought neceſ- " ſary. On the thirteenth every bad ſymptom " had entirely left her, and ſhe recovered " without any farther interruption.

" CASE IV.

" Suſan Cheetham, of Aſhton under-Line, " fourteen years of age, had the misfortune of " a compound fracture of the fore-arm, by a " fall on the 27th of June, 1782, which was " bandaged by a country ſurgeon. She was " brought to the Mancheſter Infirmary on the " 29th, and, though only two days after the " accident, her arm was completely mortified " almoſt as high as the ſhoulder, and the ſwel- " ling and inflammation had extended even " ſtill farther, and ſeemed to be progreſſive. " The mortification and other bad ſymptoms

" had

" had made fuch a progrefs, that there was
" little expectation fhe could live many hours.
" Of all mortifications, indeed, none are per-
" haps fo often fatal, or advance with fuch
" rapidity, as thofe occafioned by compound
" fractures of the fore-arm. Under fuch pref-
" fing circumftances, no time was to be loft.
" She took immediately twenty drops of the
" tinct. theb., and foon afterwards a bolus,
" containing fix grains of mufk and three
" grains of falt of hartfhorn. The bolus was
" repeated every three hours. Orders were
" alfo given for the opiate to be repeated as
" often as it was neceffary to procure eafe and
" fleep, but fhe had no occafion for it, the
" mufk and falt of hartfhorn anfwering every
" purpofe of that fort. The next morning
" the mortification feemed to have made no
" farther progrefs; fhe was perfectly com-
" pofed; her pulfe only one hundred; and the
" bolus appeared to have agreed with her fo
" well, that ten grains of mufk and fix grains
" of falt of hartfhorn were ventured on every
" three hours. She took, by this means, eigh-
" ty grains of mufk and forty-eight grains of
" falt of hartfhorn every day, and, though a
" tender and delicate girl, they were fo far

" from heating her, that every bad ſymptom " of fever gradually ceaſed. The decoction " of the bark was then directed; but ſhe diſ- " liked the taſte ſo much, that ſhe took very " little of it. In about ten days the mortifi- " cation was not only ſtopped, but far ad- " vanced in its ſeparation, and every other diſ- " agreeable apprehenſion vaniſhed. The bo- " lus was in conſequence only given twice each " day, and ſhe was prevailed upon to take the " bark, as being leſs expenſive. On this " change of medicine every bad ſymptom " ſpeedily returned; her pulſe became as quick " as ever, and her delirium and loſs of ſleep " were equally as troubleſome. The bark was, " therefore, thrown aſide, and recourſe again " had to the bolus every three hours, which " ſoon produced the very favourable appea- " rances that had before attended it. She " continued the uſe of it till ſhe had taken " two ounces of muſk and nine drachms of " ſalt of hartſhorn. She then ſeemed to have " no farther occaſion for the medicine, and ſhe " has been well ever ſince. The fore-arm " dropped off at the elbow, and the os humeri " was ſawn off above the middle of the bone.

" From

" From this cafe I apprehend the efficacy of
" this medicine may be clearly inferred. The
" relief of the child's complaints when the
" mufk and falt of hartfhorn were adminifter-
" ed, the return of them when they were
" omitted, and their vanifhing again as foon
" as fhe began to take them a fecond time, are
" the cleareft proofs in its favour. It is im-
" poffible to wifh for a cafe in which its powers
" and operations can be more furely traced.
" From the moment when it was firft taken its
" effects became diftinctly vifible, and the dif-
" order was at once checked in its progrefs:
" on its temporary omiffion the patient imme-
" diately relapfed, and the complaint returned
" with all its former vigour. The medicine
" was again applied to with the fame happy
" confequences, the diforder was a fecond time
" overpowered by its virtues, and a complete
" cure was obtained."

IX. *Patho-*

